# Encapsulated Primary Human Ovarian Cancer Cells on Chips for Chemotherapy Drug Evaluation

**DOI:** 10.34133/research.1313

**Published:** 2026-07-09

**Authors:** Qi Yang, Rui Liu, Bin Kong, Yuyang Zhang, Miaoqing Zhao, Yunlang Cai

**Affiliations:** ^1^ Department of Obstetrics and Gynecology, Zhongda Hospital, School of Medicine, Southeast University, Nanjing 210009, China.; ^2^ Department of Rheumatology and Immunology, Nanjing Drum Tower Hospital, Affiliated Hospital of Medical School, Nanjing University, Nanjing 210008, China.; ^3^ Department of Biomedical Engineering, School of Medicine, Shenzhen University, Shenzhen, Guangdong 518000, China.; ^4^ Department of Gynecology, The First Affiliated Hospital of Wenzhou Medical University, Wenzhou 325000, China.; ^5^ Department of Pathology, Shandong Cancer Hospital and Institute, Shandong First Medical University and Shandong Academy of Medical Sciences, Jinan 250117, China.

## Abstract

Establishing physiologically relevant in vitro tumor models is critical for accurately evaluating chemotherapeutic efficacy in ovarian cancer. However, generating high-throughput, uniform, and viable tumor spheroids from primary patient cells remains a major challenge for personalized drug screening. Here, we present an integrated platform that couples hydrogel microencapsulation with organ-on-a-chip culture to achieve biomimetic 3-dimensional tumor growth and dynamic drug assessment. Core–shell hydrogel microcapsules, consisting of a carboxymethyl cellulose core and an alginate shell, create a biocompatible and tunable microenvironment that maintains cell viability, preserves molecular and phenotypic heterogeneity, and enables reproducible spheroid formation. Embedding these spheroids into a microfluidic chip with continuous, precisely regulated drug gradients and perfusion culture facilitates high-resolution, high-throughput evaluation of chemotherapeutic responses. Using this system, clinically relevant agents—including carboplatin, paclitaxel, docetaxel, and pegylated liposomal doxorubicin—were systematically screened, revealing patient-specific variations in drug sensitivity that closely aligned with postoperative clinical outcomes. By integrating hydrogel-based 3-dimensional culture with microscale gradient control, this approach provides a stable, physiologically meaningful, and scalable platform for preclinical pharmacological testing. Collectively, the findings demonstrate its potential as an effective tool for individualized chemotherapy evaluation and precision treatment development in ovarian cancer.

## Introduction

Ovarian cancer (OC) is the most lethal gynecologic malignancy, with approximately 230,000 new diagnoses annually worldwide [1]. Long-term prognosis for patients diagnosed at advanced stages remains extremely poor, with 5-year survival rates reported at approximately 29% [2]. This high mortality is primarily driven by 2 key factors: delayed diagnosis [3] and chemotherapy resistance [4]. Currently, primary debulking surgery followed by systemic chemotherapy represents the standard treatment for advanced OC [5,6]. However, despite the availability of long-term maintenance therapies, 70% to 80% of patients experience tumor recurrence and subsequently develop resistance to first-line chemotherapeutic agents, including platinum-based compounds and paclitaxel (PTX) [7,8]. The inherent heterogeneity of OC [9], along with variability in individual patient responses to treatment [3], presents substantial challenges in achieving truly personalized therapy based solely on clinical guidelines and experience [10,11]. Therefore, the development of in vitro culture systems that support tumor cell growth in a physiologically relevant 3-dimensional (3D) context, coupled with the exploration of tumor heterogeneity, is crucial [12–14]. Additionally, establishing an efficient drug evaluation platform capable of personalized simulation of the internal drug response in OC using primary cells is essential for advancing precision oncology and tailoring therapeutic strategies [15,16].

**Fig. 1.**
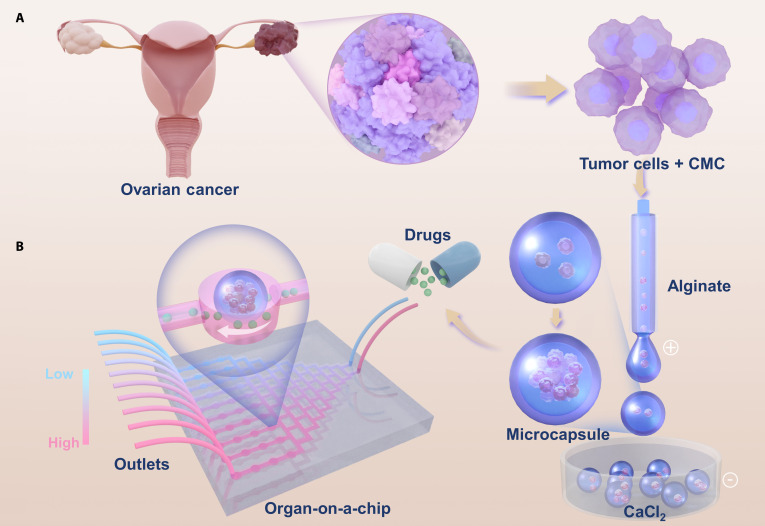
Schematic diagram of encapsulated primary human ovarian cancer (OC) cell microcapsules for chemotherapeutic drug evaluation. (A) Extraction of primary OC cells. (B) Preparation of encapsulated tumor sphere hydrogel microcapsules and integrated drug evaluation microarrays.

Here, we present a novel integrated platform that combines hydrogel microencapsulation with an organ-on-a-chip system to generate uniform and viable ovarian tumor spheroids for high-throughput chemotherapeutic screening, as illustrated in Fig. 1. Three-dimensional tumor spheroids have become increasingly valuable in drug evaluation owing to their biomimetic features [17,18] and their capacity to recapitulate the intricate interactions between cancer cells and their surrounding microenvironment [19–21]. Nevertheless, conventional spheroid formation techniques are often inefficient, yielding aggregates of inconsistent size and architecture, which diminishes their ability to accurately model patient-specific tumor heterogeneity [22]. Microfluidic electrospraying provides a robust alternative, enabling continuous fabrication of hydrogel microcapsules with precisely tunable dimensions and microstructures [23,24], thereby establishing a miniaturized and biocompatible niche for in vitro 3D culture [25,26]. Meanwhile, organ-on-a-chip technology employs microchannels and biocompatible materials to precisely regulate microscale fluid dynamics, effectively recreating organ-level physiological cues and enabling dynamic, high-throughput drug testing [27–30]. By integrating these complementary approaches, the platform combines the structural support of hydrogel microencapsulation with the dynamic perfusion and gradient control of organ-on-a-chip systems. This synergistic design provides a stable 3D microenvironment together with controlled, physiologically relevant drug delivery and enabling reproducible evaluation of chemotherapeutic responses. Importantly, this combinatorial strategy—still rarely applied to OC drug screening—establishes a versatile framework that bridges biomimetic tumor modeling and microscale pharmacological testing, offering a promising platform for personalized chemotherapy evaluation and the advancement of precision oncology.

To achieve this, primary human OC cells were encapsulated in core–shell structured hydrogel microcapsules, fabricated via coaxial microfluidic electrospray. Each microcapsule consisted of a carboxymethyl cellulose (CMC) core surrounded by an alginate (ALG) shell, forming a distinct dual-layer configuration. This hierarchical structure creates a balanced microenvironment that supports long-term culture under continuous perfusion, enhancing both the reproducibility and physiological relevance of the model. Within this microenvironment, OC cells proliferate efficiently and self-organize into uniformly sized, viable 3D tumor spheroids. By further incorporating these microcapsules into a microfluidic platform featuring a concentration gradient generator and perfusion culture chamber, we established a physiologically relevant OC model suitable for dynamic, high-throughput drug testing. Using this platform, we screened commonly used clinical chemotherapeutic agents, including carboplatin (CBP) [31], PTX [32], docetaxel (DTX) [33], and pegylated liposomal doxorubicin (PLD) [34], with results highly consistent with clinical efficacy. The platform demonstrates high reproducibility and accuracy, enabling dynamic, high-throughput evaluation of different chemotherapeutic regimens. This system holds great promise as an efficient platform for chemotherapy screening.

## Results and Discussion

**Fig. 2.**
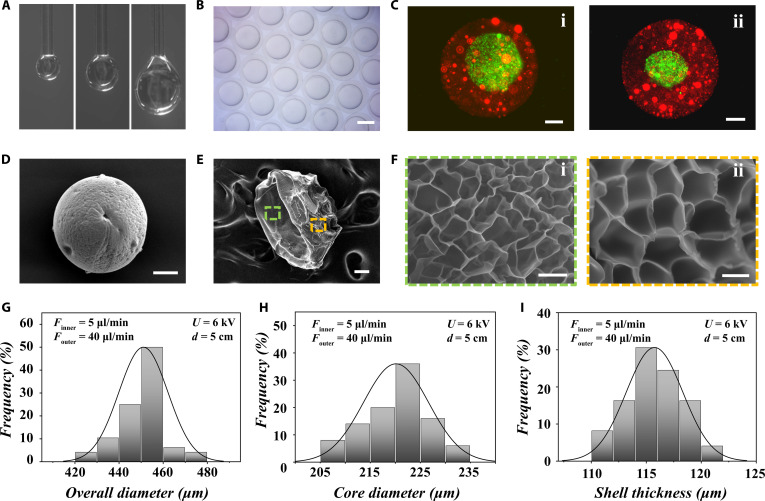
Preparation and characterization of alginate (ALG)/carboxymethyl cellulose (CMC) microcapsules. (A) Real-time droplet generation during coaxial electrospray. (B) Bright-field images of core–shell microcapsules. (C) Fluorescence images of microcapsules fabricated at outer-to-inner flow rate ratios of 10:1 (i) and 20:1 (ii). (D) Scanning electron microscopy (SEM) images of intact and (E) sectioned microcapsules. (F) High-magnification SEM images of the porous microstructures in the core (i) and shell (ii). (G to I) Size distribution analysis under optimized fabrication conditions: (G) overall capsule diameter, (H) core diameter, and (I) shell thickness. Scale bars are 200 μm in (B), 100 μm in (C), 50 μm in (D) and (E), and 5 μm in (F).

In this study, uniform core–shell hydrogel microcapsules with excellent biocompatibility were fabricated using a microfluidic electrospray technique. A custom-designed coaxial capillary microfluidic device (Fig. S1) was constructed to enable precise control over droplet generation. During operation, the outer ALG phase encapsulated the inner CMC phase through hydrodynamic focusing, after which the coaxial streams were subjected to electrospray, producing monodisperse droplets (Fig. 2A) that were immediately collected in a 2% CaCl_2_ solution, where rapid ionic cross-linking induced gelation of the ALG shell, thereby forming core–shell hydrogel microcapsules. The resulting configuration (Fig. 2B) offered both mechanical integrity and a favorable microenvironment for subsequent 3D cell culture. By tuning the core-to-shell flow rate ratio, the shell thickness and internal cavity could be precisely modulated. To visualize the spatial distribution of the 2 phases, fluorescent nanoparticles, red in ALG (shell) and green in CMC (core), were incorporated as tracers. After static incubation in phosphate-buffered saline (PBS) for 11 d, the green fluorescence remained confined to the core region (Fig. 2C), confirming the persistence of CMC and the structural stability of the core–shell microcapsules throughout the culture period.

**Table 1.** Clinical information for patients with OC corresponding to the 3 kinds of tumor spheroids

Patient	Age	Histological type	FIGO stage	WHO grading	Chemotherapy
Sample 1	52	Serous adenocarcinoma	IIB	Low grade	TC
Sample 2	49	Serous adenocarcinoma	IIA	High grade	TC
Sample 3	51	Serous adenocarcinoma	IIA	Low grade	TC

OC, ovarian cancer; FIGO, International Federation of Gynecology and Obstetrics; WHO, World Health Organization; TC, paclitaxel plus carboplatin

To achieve microcapsules with uniform morphology, controlled size, and structural integrity, key fabrication parameters were systematically investigated. As shown in Fig. S2a to d, the internal (*F*_inner_) and external (*F*_outer_) flow rates, applied voltage (*U*), and spray distance (*d*) collectively governed droplet formation and the resulting microcapsule dimensions. Increasing *F*_inner_ enlarged both the overall capsule diameter and the core size, whereas increasing *F*_outer_ at a constant *F*_inner_ slightly increased the capsule diameter while reducing the core size. Scanning electron microscopy (SEM) confirmed the well-defined core–shell architecture of the microcapsules (Fig. 2D and E) and revealed interconnected porous structures within both the shell and core regions (Fig. 2F), which facilitate nutrient diffusion and metabolic waste exchange. Under optimized conditions, the microcapsules exhibited a uniform size distribution with an average diameter of approximately 450 μm, a core diameter of 220 μm, and a shell thickness of 115 μm (Fig. 2G to I), providing sufficient internal space and structural stability for tumor spheroid formation.

Furthermore, mechanical and stability analyses were performed to assess their physicochemical robustness. Compression testing showed that hydrogel stiffness increased with ALG concentration, with 2.5 wt% ALG exhibiting the highest stress values (Fig. S3). Considering that excessive rigidity may compromise microcapsule handling and cellular microenvironment adaptability, 1.5 wt% ALG was selected. In addition, swelling analysis in PBS revealed a moderate increase during the initial incubation period followed by stabilization (Fig. S4a), while degradation assays showed only a gradual mass loss over the 15-d culture period without structural collapse, as shown in Fig. S4b. Collectively, these results confirm that the optimized core–shell microcapsules possess stable mechanical properties and long-term structural integrity suitable for sustained 3D culture and dynamic drug screening applications.

**Fig. 3.**
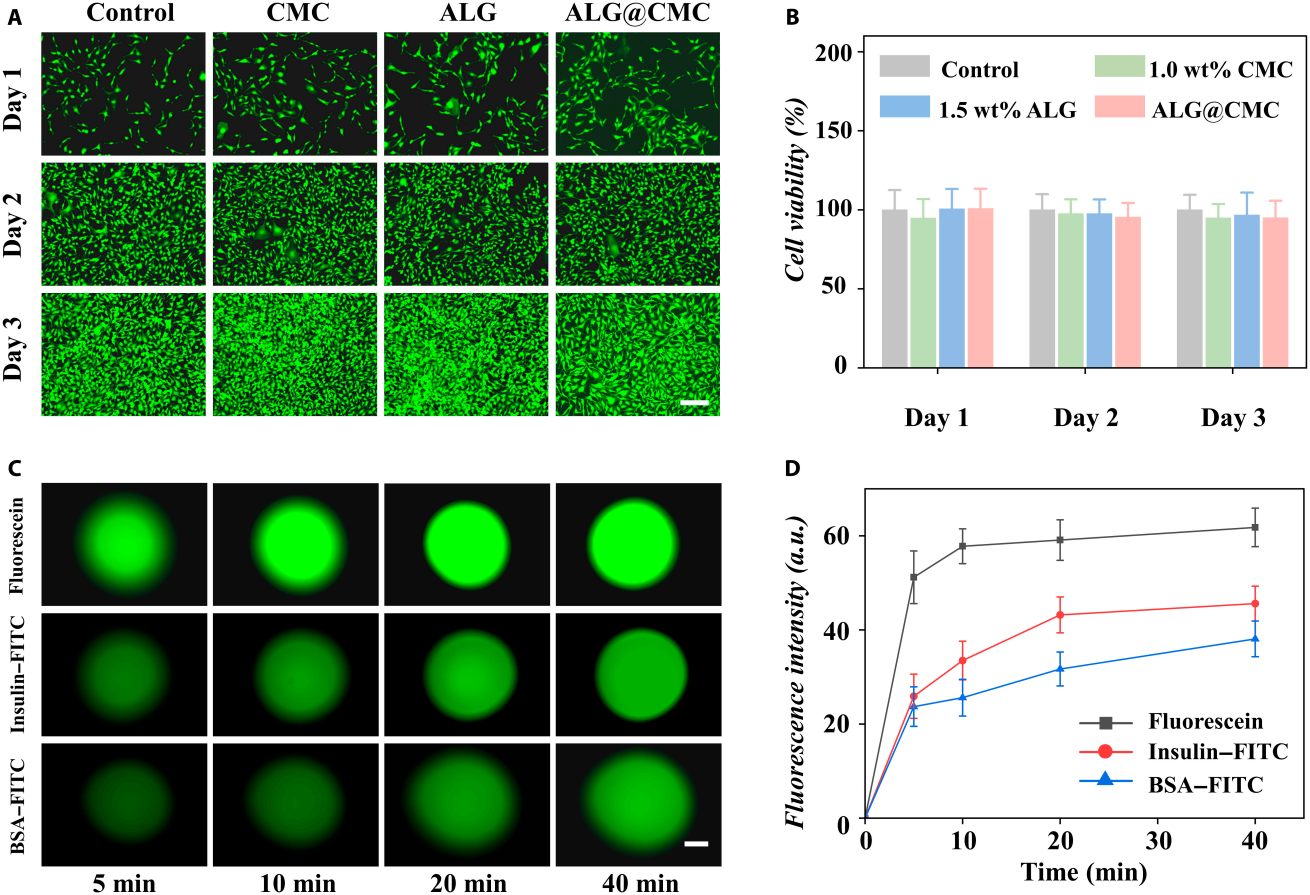
Cytocompatibility and permeability characterization of alginate (ALG)/carboxymethyl cellulose (CMC) microcapsules. (A) Fluorescence images of primary ovarian cancer (OC) cells cultured with ALG/CMC, ALG, or CMC extracts. (B) Quantitative cell viability over 3 d. (C) Fluorescence images showing diffusion of tracers with different molecular weights into microcapsules. (D) Quantitative fluorescence intensity profiles corresponding to tracer diffusion in microcapsules. Scale bars are 200 μm in (A) and 50 μm in (C).

The biocompatibility of the fabricated microcapsules was assessed using an extract-based cytotoxicity assay with primary OC cells. After 72 h of incubation with the microcapsule extracts, the cells retained normal morphology and exhibited robust proliferative capacity (Fig. 3A). Cell Counting Kit-8 assay results further confirmed that cell viability remained above 90% across all treated groups (Fig. 3B), indicating minimal cytotoxicity and excellent cytocompatibility. Moreover, the molecular permeability of the microcapsules was systematically assessed using fluorescently labeled molecules of different molecular weights (MWs). Fluorescein (MW 376 Da) rapidly diffused into the microcapsules, while insulin–fluorescein isothiocyanate (FITC) (MW 5.8 kDa) and bovine serum albumin (BSA)–FITC (MW 66.0 kDa) displayed slower diffusion kinetics but achieved stable fluorescence within 40 min (Fig. 3C and D). These findings demonstrate that the microcapsules possess favorable permeability properties, sufficient to support nutrient exchange and metabolic waste removal during long-term cell culture.

**Fig. 4.**
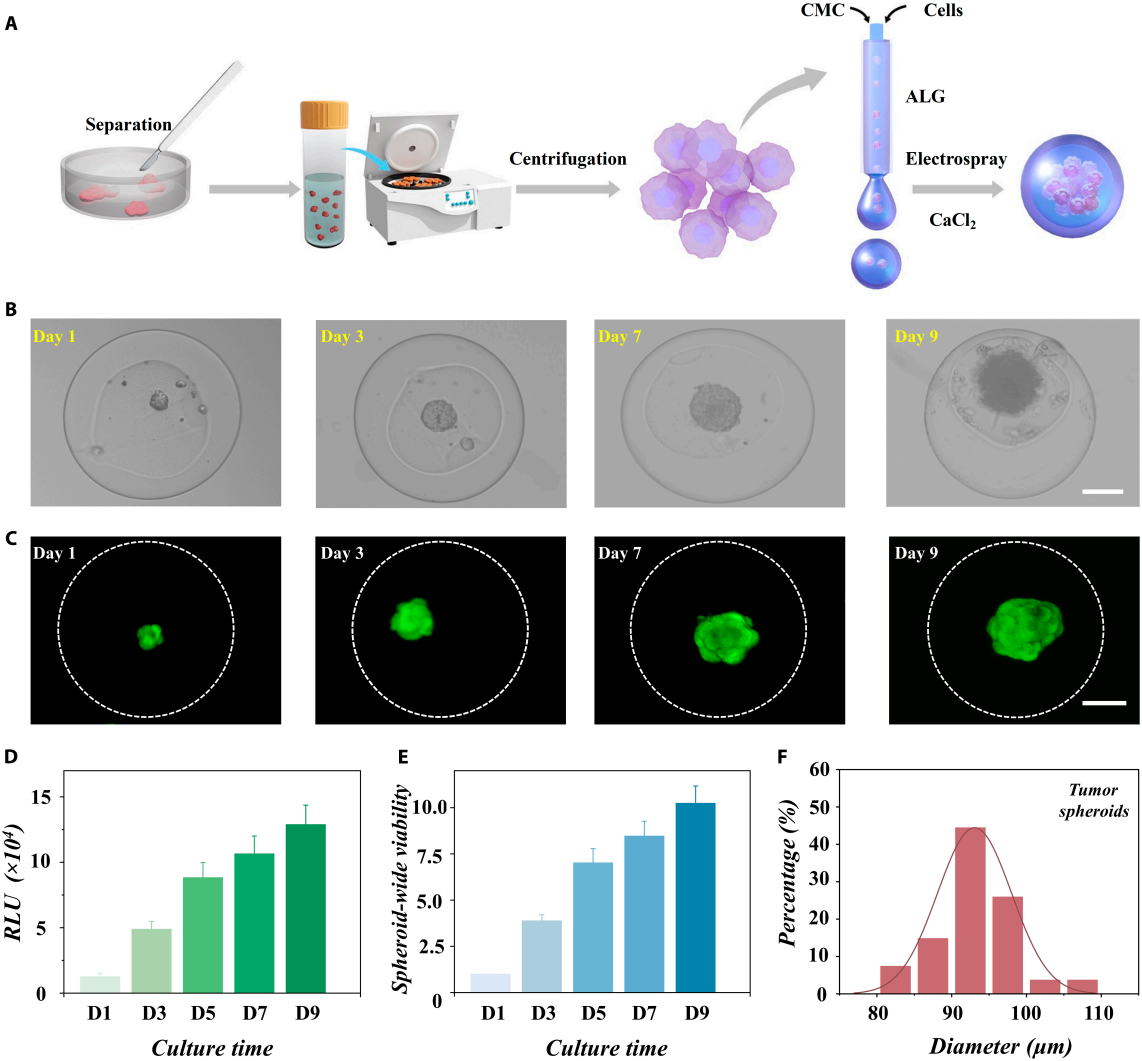
Formation and characterization of tumor spheroids within hydrogel microcapsules. (A) Schematic illustration of primary ovarian cancer (OC) cell isolation and encapsulation using electrospray microfluidics. (B) Bright-field images showing spheroid formation within microcapsules on days 1, 3, 7, and 9. (C) Live/dead staining of encapsulated tumor spheroids at corresponding time points. (D) Quantification of spheroid proliferation over time. (E) Viability assessment of spheroids during culture. (F) Size distribution of tumor spheroids on day 9. Data are presented as mean ± SD. Scale bars in (B) and (C) are 100 μm.

To evaluate the feasibility of 3D tumor spheroid culture within microcapsules, primary OC tissues obtained from patients were processed to establish patient-derived spheroid cultures. Briefly, the tumor specimens were mechanically minced into small fragments and enzymatically dissociated into single-cell suspensions using collagenase digestion (Fig. S5a to c). In the study, 3 patient-derived samples were processed, and spheroids were successfully established from all samples, which were subsequently used for downstream drug screening analyses. The resulting cells were mixed with CMC solution as the internal phase and subsequently encapsulated using the optimized microfluidic electrospray protocol to generate cell-laden porous microcapsules (Fig. 4A and Fig. S5d). The obtained microcapsules were subsequently maintained in complete culture medium at 37 °C in a humidified incubator with 5% CO_2_ and 95% air atmosphere. Primary OC cells began to spontaneously aggregate within the microcapsules as early as day 1, forming small clusters (Fig. S5e to h). With prolonged culture, these clusters progressively enlarged and developed into compact spheroids with well-defined boundaries and sustained viability (Fig. 4B). Calcein-AM/propidium iodide (PI) staining and proliferation analysis further confirmed high cell viability and active proliferation during culture (Fig. 4C and D). Growth kinetics showed progressive spheroid expansion up to day 9 while maintaining high viability (Fig. 4E), demonstrating the excellent biocompatibility of the core–shell microcapsules for supporting 3D tumor spheroid formation. Notably, spheroid size distribution on day 9 exhibited high uniformity and a narrow range (Fig. 4F), indicating the good reproducibility of the microencapsulation-based culture system. Collectively, these results demonstrate that the microcapsule platform enables stable and reproducible generation of patient-derived tumor spheroids, providing a reliable basis for subsequent drug screening studies.

**Fig. 5.**
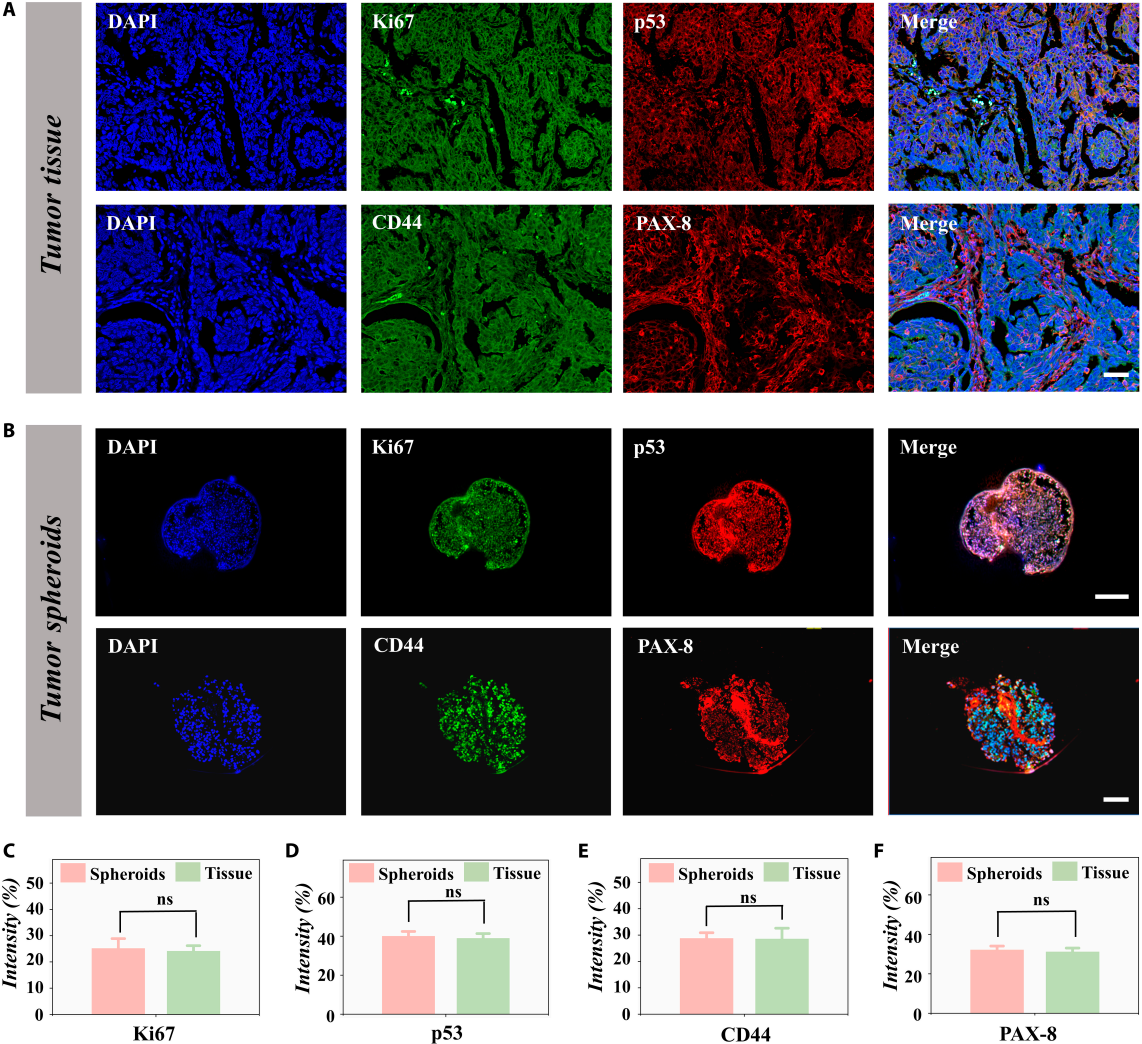
Comparison of molecular marker expression in ovarian cancer (OC) tissues and microencapsulated tumor spheroids. (A) Immunofluorescence images of OC tissues stained for Ki67, p53, CD44, and PAX-8. (B) Corresponding staining of tumor spheroids generated in microcapsules. (C to F) Quantitative of fluorescence intensity for Ki67 (C), p53 (D), CD44 (E), and paired box gene 8 (PAX-8) (F) in tissue and spheroids (*n* = 3). Scale bars are 50 μm in (A) and 50 μm in (B).

The retention of the key molecular features of primary OC in tumor spheroids generated through microencapsulation was evaluated using immunofluorescence and immunohistochemical analyses targeting lineage, tumor-associated, and proliferation markers. Hematoxylin and eosin (H&E) staining was first performed on adjacent serial sections derived from the same tissue blocks (Fig. S6). The H&E images revealed the typical histoarchitectural features of OC and enabled clear discrimination between tumor and stromal regions, thereby providing a reliable morphological reference for subsequent marker-based analyses. Primary OC tissues were first evaluated for paired box gene 8 (PAX-8), p53, CD44, and Ki67 expression (Fig. 5A and Fig. S7). Spheroids derived from microencapsulated cultures were subsequently stained using the same antibody panel to assess their molecular concordance with the original tissues. PAX-8, a lineage marker of epithelial ovarian carcinoma, served to confirm tumor origin [35]. p53, a tumor-associated marker in ovarian carcinoma, was included to characterize tumor-related molecular features [36]. CD44, a broadly expressed cell-surface adhesion molecule in ovarian epithelial tumors [37], and Ki67, a proliferation-associated and prognostic marker [38], were employed to assess tumor aggressiveness. Immunostaining revealed that the microencapsulated spheroids retained the overall expression patterns of these markers observed in the corresponding patient tissues (Fig. 5B), as expected for a 3D in vitro model. Quantitative fluorescence analysis further supported the general concordance in marker expression profiles between tissues and spheroids (Fig. 5C to F). Notably, integration of histomorphological assessment with immunohistochemical findings indicated that the p53 staining pattern was consistent with a wild-type expression profile. Together, these results indicate that spheroids generated through microencapsulation preserve the key molecular features of the parental tumors, supporting their suitability as a physiologically relevant in vitro model for subsequent chemotherapeutic evaluation.

**Fig. 6.**
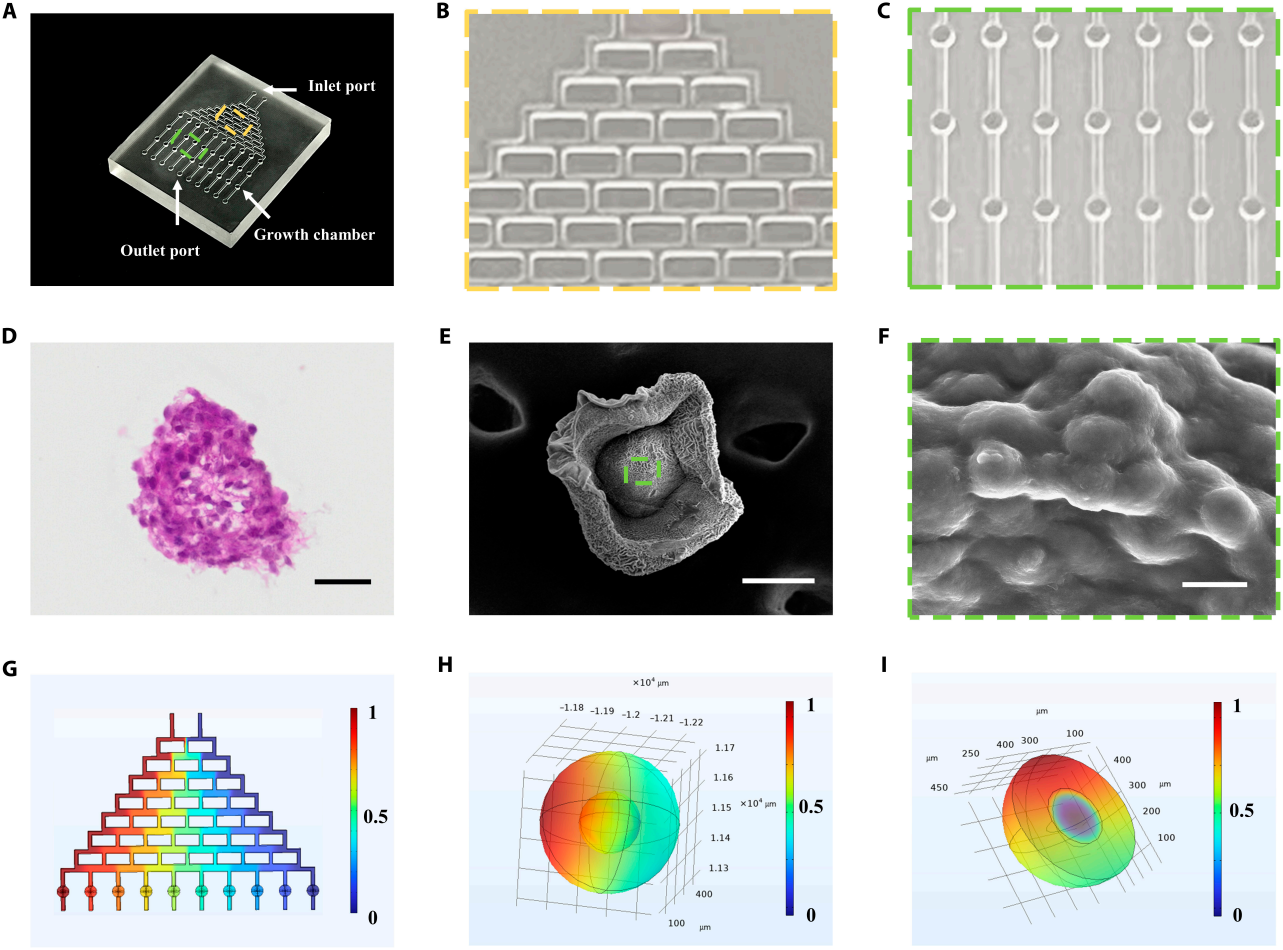
Microfluidic platform for tumor spheroid culture and gradient generation. (A) Photograph of the microfluidic drug screening chip. (B) Optical micrograph of the concentration gradient generator. (C) Optical micrograph of the spheroid culture chamber. (D) Hematoxylin and eosin (H&E) staining of an ovarian cancer (OC) spheroid. (E) Scanning electron microscopy (SEM) image of the tumor spheroid and (F) higher-magnification view of the spheroid surface. (G) Numerical simulation of concentration gradient formation within the gradient generator. (H) Simulation of drug diffusion into tumor-laden microcapsules (the *x*-axis indicates flow direction). (I) Cross-sectional simulation showing drug penetration into the tumor spheroid. Scale bars are 50 μm in (D),100 μm in (E), and 10 μm in (F).

To mimic physiologically relevant drug diffusion and enable high-throughput pharmacological assessment, a microfluidic chip integrating a dendritic concentration gradient generator with a multichamber culture module was designed and fabricated (Fig. 6A). In this platform, each chip contains multiple independent culture chambers that can be operated simultaneously, allowing parallel testing of different drug conditions under identical perfusion settings. Microcapsules containing primary OC spheroids were carefully loaded into the individual culture chambers and maintained under continuous perfusion. The culture medium was introduced through the inlet and distributed across the chambers via the gradient generator (Fig. 6B and C), establishing a controlled microenvironment that ensured stable oxygen and nutrient supply while facilitating metabolic waste removal. Compared with static culture, this dynamic perfusion system better preserved cell viability and supported the sustained maintenance of 3D tumor spheroids. On day 3, H&E staining and SEM were performed to characterize spheroid morphology and internal architecture. H&E staining revealed well-organized tissue-like structures (Fig. 6D), whereas SEM images showed compact spheroid surfaces and preserved structural integrity (Fig. 6E and F), demonstrating that the microfluidic environment maintained stable 3D growth under continuous flow. Collectively, these results confirm that the established microfluidic culture platform enables consistent maintenance of viable, morphologically intact tumor spheroids, providing a reliable and reproducible in vitro system for subsequent drug testing and mechanistic studies.

The therapeutic efficacy of antitumor agents depends on their ability to achieve and maintain effective concentrations within the tumor microenvironment. To evaluate the capability of the microfluidic chip to generate stable concentration gradients and determine appropriate flow conditions, rhodamine B and methylene blue solutions were introduced through the 2 inlets using a constant-flow syringe pump at flow rates of 1, 2.5, and 5 μl/min. At 2.5 μl/min, the dyes mixed efficiently at each bifurcation of the dendritic gradient generator, producing a stable and continuous gradient along the downstream channels (Fig. S8a to c). To validate the generation of concentration gradients and assess drug diffusion behavior, numerical simulations using COMSOL Multiphysics, based on the Navier–Stokes and convection–diffusion equations, further confirmed the formation of a stable linear concentration gradient across the microfluidic channels (Fig. 6G, Fig. S9a, and Movie S1). Under the same flow conditions, further simulations demonstrated that drug molecules diffused efficiently from the surrounding medium into the microcapsules and subsequently penetrated the tumor spheroids, achieving a relatively uniform intratumoral distribution, as shown in Fig. 6H and I, Fig. S9b and c, and Movies S2 and S3. Importantly, the chip architecture contains multiple parallel culture chambers that can be operated simultaneously, allowing parallel evaluation of different drug concentrations under identical perfusion conditions. Collectively, these results indicate that the microfluidic platform reliably supports stable gradient formation and controlled solute diffusion under physiologically relevant flow conditions, providing a robust and reproducible basis for subsequent high-throughput drug screening in primary tumor spheroids.

To evaluate the applicability of the tumor-spheroid-loaded microfluidic platform for in vitro chemotherapeutic screening, patient-derived tumor cells from 3 OC patients were encapsulated within hydrogel microcapsules and cultured for 9 d before drug screening, yielding 3 models designated as samples 1 to 3. Flow cytometry analysis confirmed comparable baseline apoptotic levels among the samples (Fig. S10). The microencapsulated spheroids were then loaded into individual incubation chambers of the microfluidic chip and exposed to a panel of clinically relevant chemotherapeutic regimens, including 4 commonly used single agents, CBP, PTX, DTX, and PLD, as well as representative sequential combinations reflecting clinical treatment strategies (Fig. S11).

**Fig. 7.**
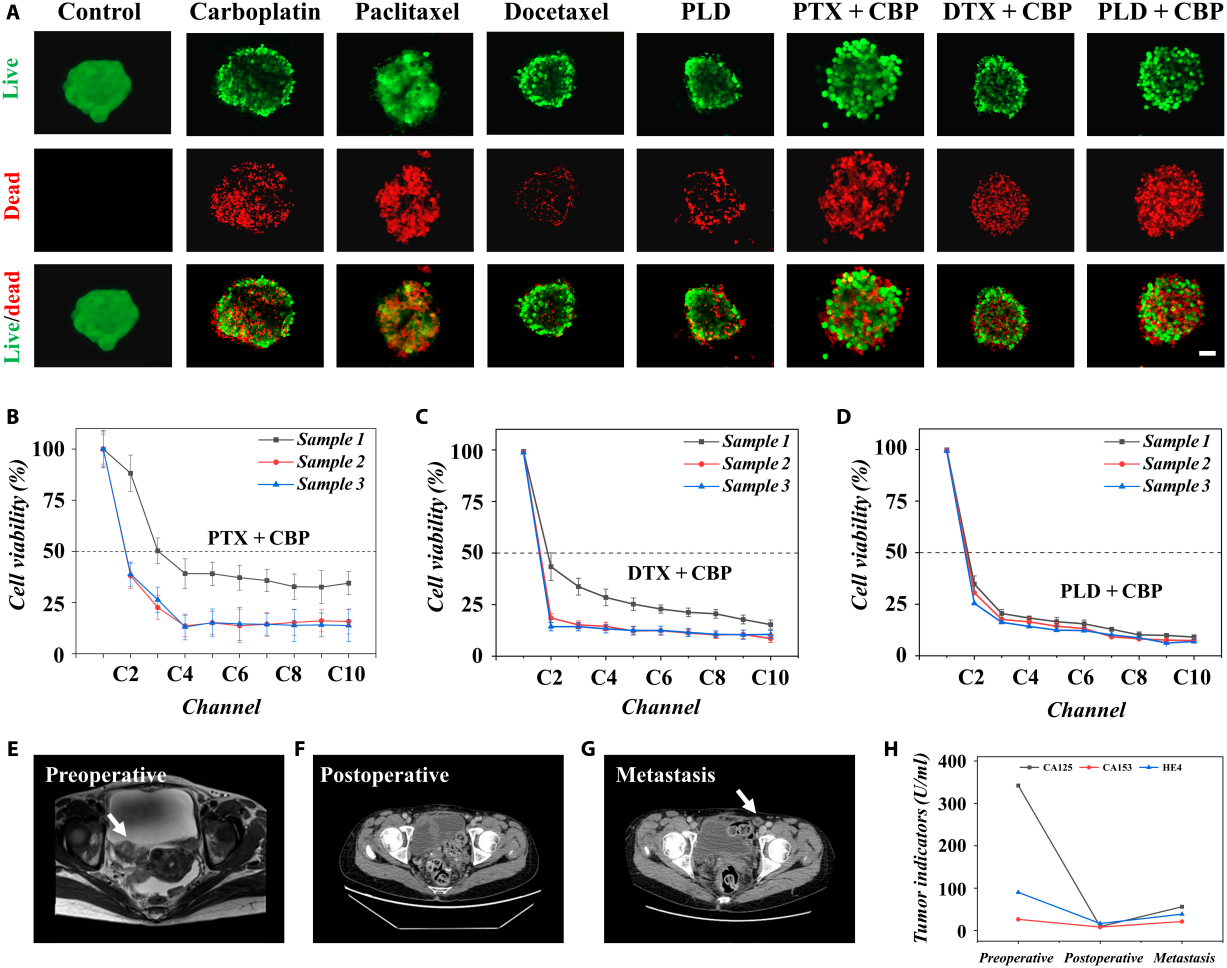
Drug response of patient-derived tumor spheroids and corresponding clinical imaging. (A) Confocal images of tumor spheroids from sample 1 after treatment with 7 chemotherapeutic regimens, assessed by live/dead staining. (B to D) Quantification of tumor spheroids 72 h posttreatment with different drug combinations. (E to G) MRI images of the sample 1 patient at 3 clinical stages: (E) preoperative, (F) postoperative, and (G) metastatic progression following the third chemotherapy cycle. (H) Tumor metrics of sample 1 patients across the corresponding clinical stages. The scale bar is 20 μm in (A).

Tumor spheroids derived from sample 1 were first subjected to the complete treatment panel, and therapeutic responses were visualized by calcein-AM/PI staining (Fig. 7A). Distinct viability distributions were observed among treatment groups, reflecting the heterogeneous chemosensitivity of the patient-derived tumor cells. After 72 h of continuous single-agent exposure, overall viability remained comparable across all samples (Fig. S12a to d). However, sequential administration of PTX followed by CBP, mirroring the standard first-line regimen, elicited a more resistant phenotype in sample 1 relative to samples 2 and 3 (Fig. 7B). Other combination treatments yielded more uniform responses across patient-derived models (Fig. 7C and D). Collectively, these results demonstrate that the microfluidic system sustains the physiological activity and individualized drug response profiles of ovarian tumor spheroids, offering a reproducible and physiologically relevant platform for assessing chemotherapeutic efficacy and interpatient variability under controlled microenvironmental conditions. Moreover, the multichamber architecture of the microfluidic chip enables parallel testing of multiple treatment conditions within a single device, facilitating efficient drug screening of patient-derived tumor spheroids.

Notably, postoperative follow-up revealed that the patient corresponding to sample 1 developed recurrence and distant metastasis (left inguinal region) after 3 cycles of combination chemotherapy with PTX and CBP (Fig. 7E to G), accompanied by an increase in tumor-associated serological markers (Fig. 7H). Importantly, optimal cytoreductive surgery with R0 resection was achieved at the time of primary surgery, and no metastatic lesions were detected on postoperative imaging. In contrast, the patients corresponding to samples 2 and 3 exhibited no evidence of recurrence or metastasis after completing 6 cycles of the same regimen. These observations suggest that the tumor spheroids retained, at least in part, the intrinsic biological heterogeneity of their parental tumors. Histopathological examination revealed distinct clinicopathological features among the 3 patient samples (Table 1). Although samples 1 and 3 were both classified as low-grade serous ovarian carcinoma, their tumor spheroids exhibited different chemotherapy responses. Notably, sample 1 showed reduced chemosensitivity compared with samples 2 and 3, reflecting chemoresistant tendency and interpatient heterogeneity. Together, these findings indicate that the integrated platform combining microencapsulated tumor spheroids with a microfluidic gradient system can partially recapitulate patient-specific therapeutic responses in vitro. Although the present study is limited by the small number of patient-derived samples and the histological heterogeneity of the included cases, the qualitative concordance between in vitro drug response patterns and the corresponding clinical outcomes suggests the potential feasibility of this platform for individualized chemotherapy evaluation. Further validation in a larger and more histologically representative sample set will be necessary to determine its broader applicability.

## Conclusion

In summary, we developed an integrated organ-on-a-chip platform that combines hydrogel microencapsulation with dynamic microfluidic culture to enable physiologically relevant modeling and chemotherapeutic evaluation of OC. The core–shell microcapsules provide a stable 3D microenvironment that supports the formation of viable patient-derived tumor spheroids, while the microfluidic gradient system enables controlled and dynamic drug exposure. This platform allows parallel assessment of drug responses in tumor spheroids while maintaining the key features of tumor heterogeneity. Notably, the qualitative agreement between in vitro drug responses and corresponding clinical outcomes suggests the potential of this system to capture patient-specific therapeutic variability. Although further validation in larger and more representative patient-derived samples is required, the integrated microcapsule–microfluidic platform described here provides a scalable and physiologically relevant framework for preclinical drug evaluation in OC.

## Methods

### Materials

ALG (high viscosity), CMC (low viscosity), calcium chloride, fluorescein (MW 376 Da), insulin–FITC (MW 5.8 kDa), BSA–FITC (MW 66.0 kDa), glutaraldehyde, dimethyl sulfoxide, and ALG lyase were sourced from Sigma-Aldrich. The fluorescent nanoparticles (L4655 and L3280) and antibodies were from Thermo Fisher. Collagenase II and TrypLE (Gibco) were used for primary cell isolation. Live/dead staining (Beyotime) and CellTiter-Glo kits (Promega) assessed cell viability. Anticancer drugs (PTX, CBP, PLD, and DTX) were from McLean and prepared at working concentrations. Polydimethylsiloxane (Sylgard 184, Dow Corning) was used for microfluidic mold fabrication.

### Microfluidic electrospray setup and microcapsule fabrication

A microfluidic electrospray system was constructed by integrating a capillary with a high-voltage electrospray module. At the flow junction, the inner phase (1 wt% CMC) formed a stable laminar stream, hydrodynamically focused and encapsulated by the outer phase (1.5 wt% ALG) due to the low Reynolds index. Upon application of a high-voltage electric field, the coaxial jet disintegrated into droplets and was directed into a CaCl_2_ solution of the collection pool, where the ALG shell cross-linked to yield core–shell microcapsules. Flow rates, voltage, and collection distance were systematically varied to modulate capsule morphology.

### Mechanical characterization of hydrogels

The mechanical properties of ALG hydrogels were evaluated using a universal testing machine. Briefly, hydrogel samples were prepared as cylindrical specimens with a diameter of 5 mm and a height of 2 mm. The samples were subjected to uniaxial compression at a constant loading rate of 1 mm/min, and the applied force and displacement were recorded continuously during testing.

### Swelling ratio

The freshly prepared microcapsules were dried in an oven, and the initial weight was recorded as *W*_0_. The samples were then immersed in PBS and incubated at 37 °C. At predetermined time points, the microcapsules were collected, gently blotted to remove excess surface liquid, and weighed (*W_t_*). The swelling ratio (%) was calculated using the following equation:

$$\text{Swelling ratio}\;\left(\%\right)=\frac{W_{t}-W_{0}}{W_{0}}\times 100$$ (1)

### In vitro degradation assay

The initial weight of the samples was recorded as *W*_0_, after which the microcapsules were immersed in PBS and maintained at 37 °C. At predetermined time points, the samples were retrieved, gently dried to remove surface moisture, and weighed (*W_t_*). The mass remaining was expressed as a percentage of the initial mass and calculated as follows:

$$\text{Mass remaining}\;\left(\%\right)=\frac{W_{t}}{W_{0}}\times 100$$ (2)

### Biocompatibility assays

To assess the biocompatibility of the microencapsulation materials, the leachate approach was applied. Primary OC cells were plated into 96-well plates and cultured for 24 h to facilitate adhesion prior to extract exposure. Following removal of the medium, each extract was added, and cells were cultured for an additional 72 h. Cells without extract exposure served as controls. Viability was quantified using the Cell Counting Kit-8 assay. Furthermore, calcein-AM/PI staining was employed to differentiate live and dead cells, and fluorescence imaging was performed using an inverted fluorescence microscope (Leica) for qualitative analysis.

### Microcapsule permeability test

To investigate molecular permeability, approximately 50 microcapsules were transferred to culture dishes and individually incubated with 1 μM solutions of fluorescein (MW 376 Da), insulin–FITC (MW 5.8 kDa), and BSA–FITC (MW 66.0 kDa). The fluorescence intensity within the microcapsules was monitored at 5, 10, 20, and 40 min using an inverted fluorescence microscope (Leica), allowing evaluation of diffusion kinetics across the capsule membranes.

### Human OC specimens

Ovarian tumor specimens were obtained from patients undergoing surgical resection at the Department of Obstetrics and Gynecology, Zhongda Hospital, Southeast University (Nanjing, China). All samples were pathologically confirmed as malignant by the Department of Pathology following histological examination. Informed consent was obtained from all participants prior to surgery. The study protocol was approved by the Ethics Committee of Zhongda Hospital, Southeast University (Approval No. 2022ZDSYLL088-P01).

### Human ovarian tumor cell extraction

Human OC tissues were rinsed with PBS, minced into approximately 2-mm^3^ fragments, and enzymatically dissociated in collagenase II solution. After digestion, the cell suspension was pipetted to further dissociate aggregates and filtered through a cell strainer to remove undigested debris. The cell was resuspended in erythrocyte lysis, incubated for 3 min, and centrifuged again under the same conditions. This step was repeated until red blood cells were no longer visible. The final pellet containing primary OC cells was collected for downstream applications.

### Manufacturing of cellular microcapsules

Encapsulated primary OC cells were cultured in a medium supplemented with the following components: R-spondin 100 ng/ml, epidermal growth factor 50 ng/ml, Noggin 100 ng/ml, fibroblast growth factor-10 10 ng/ ml, *N*-acetyl l-cysteine 1.25 mM, A83-01 500 nM, nicotinamide 10 mM, Y-27632 5 μM, GlutaMAX 1×, B27 1×, heregulinβ-1 37.5 ng/ml, forskolin 10 μM, β-estradiol 100 nM, HEPES 10 mM, and penicillin–streptomycin (PS) 1%. Cell-laden microcapsules were maintained under standard culture conditions for 9 d, with medium replacement performed every 2 to 3 d. Cell growth and viability were monitored throughout the culture period.

### Cell viability assessment within microcapsules

A live/dead kit was utilized to assess the viability of primary OC cells encapsulated within the microcapsules. Microcapsules were retrieved from culture medium and incubated with staining solution. Confocal microscopy was employed to acquire fluorescence images. For quantitative analysis, cell-laden microcapsules were transferred to 96-well plates, followed by the addition of the CellTiter-Glo reagent. After shaking for 10 min, luminescence was measured with a multifunctional microplate reader.

### H&E staining of OC tissue

Patient-derived OC tissues were fixed in 10% neutral-buffered formalin, followed by dehydration through a graded ethanol series, xylene clearing, and paraffin embedding. Paraffin-embedded tissues were sectioned and then deparaffinized and rehydrated prior to staining. Sections were stained with hematoxylin to visualize nuclear morphology and subsequently counterstained with eosin to delineate cytoplasmic and extracellular components. After dehydration and clearing, the slides were mounted and examined under a bright-field microscope to evaluate histological architecture and cellular features.

### Histological and immunofluorescence analysis of tumor tissues

Fresh tissue was fixed in formalin, paraffin-embedded, sectioned, deparaffinized, and then antigenically repaired. For immunofluorescence analysis, adjacent serial sections derived from the same tissue block of the same patient were used to ensure consistency across staining. Circles were drawn on the sections with an immunohistochemical pen. The sections were then immunohistochemically stained with Ki67 (1:100), PAX-8 (1:200), CD44 (1:500), and p53 (1:500).

### Immunofluorescence staining of ovarian tumor spheroids

Tumor spheroids were treated with 4% paraformaldehyde for 2 h to fix, followed by permeabilization and serum blocking for 1 h. Spheroids were incubated overnight at 4 °C with primary antibodies, followed by 1-h incubation with fluorescent secondary antibodies, 4′,6-diamidino-2-phenylindole counterstaining, and antifade mounting. A Zeiss confocal laser scanning microscope was used to acquire fluorescence images. Spheroids were incubated with primary antibodies including Ki67 (1:100), PAX-8 (1:200), CD44 (1:500), and p53 (1:500).

### Tumor sphere apoptosis assay

Microcapsules were digested with ALG lyase to release tumor spheroids, which were then dissociated into single cells using trypsin. Cells were washed and centrifuged, and the resulting pellets were gently resuspended in binding buffer for apoptosis analysis. Apoptotic cells were quantified by flow cytometry with FlowJo VX.

### Characterization

Microcapsule structures were visualized using an optical microscope (Olympus). Tumor spheroids were preserved in 2.5% glutaraldehyde, sequentially dehydrated with graded ethanol, and metal-coated with gold–palladium for SEM. Confocal fluorescence imaging was performed to visualize spheroids, and 3D image rendering and analysis were carried out using Imaris Viewer x64.

### Design of the microfluidic chip

The microfluidic device was composed of 2 inlet ports, a density gradient generator, a circular chamber for cell spheroid incubation, and a single outlet. The microchannel featured a T-shaped configuration with dimensions of 500 μm in both width and height. The spheroid incubation chamber had a cylindrical geometry, measuring 1 mm in diameter and height.

### Chip concentration gradient formation validation

In order to verify whether the chip could form a concentration gradient, rhodamine B and methylene blue solutions were injected into the chip by the cross-flow syringe pump at flowing rates of 1, 2.5, and 5 μl/min, respectively.

### Microfluidic drug evaluation

Primary OC cell-laden microcapsules were cultured in vitro for 9 d and then individually transferred into the incubation chambers of a microfluidic chip, with one microcapsule per chamber. To mimic clinical chemotherapy strategies, 7 treatment regimens were established, including 4 single-agent therapies and 3 sequential combination protocols. For monotherapy treatments, 1 μM PTX, CBP, PLD, or DTX was continuously perfused through the left inlet, while complete culture medium was simultaneously introduced through the right inlet for 72 h. Sequential combination regimens consisted of a 4-h exposure to the first agent (PTX, DTX, or PLD), followed by 24-h perfusion of CBP and subsequently drug-free medium in both inlets for the remaining 44 h. After 72 h of treatment, microcapsules were collected from the chip, and tumor spheroids were released. Cell viability was quantified using the CellTiter-Glo Luminescent Cell Viability Assay.

### Statistical analysis

The Origin 2024 software was used to analyze the data and plot the graphs. Data are expressed as mean ± standard deviation. Statistical differences were analyzed by analysis of the Student *t* test. Significance was indicated by symbols as described in the figure legends. ns = not significant.

## Ethical Approval

The study protocol was approved by the Ethics Committee of Zhongda Hospital, Southeast University (Approval No. 2022ZDSYLL088-P01).

## Data Availability

All data are available in the main text or the Supplementary Materials.
