# Supplementary material for: Encapsulated Primary Human Ovarian Cancer Cells on Chips for Chemotherapy Drug Evaluation
Source: Research (Wash D C). 2026 Jul 9;9:1313. doi: 10.34133/research.1313 (PMC13346662; doi:10.34133/research.1313)
Supplement: Supplementary 1 — Figs. S1 to S12 Movies S1 to S3 [file research.1313.f1.zip › Revised SI (Clear Version).docx]

**Supporting Information**

**Encapsulated Primary Human Ovarian Cancer Cells on chips for Chemotherapy Drug Evaluation**

Qi Yang^1^, Rui Liu^2^, Bin Kong ^3,*^, Yuyang Zhang ^4,^*, Miaoqing Zhao^5,*^, Yunlang Cai^1,*^

**Figure S1.** Microfluidic device physical brightfield diagram.

**Figure S2.** Microcapsules with different diameters. Influence of (a) CMC flow rate, (b) ALG flow rate, (c) Voltage and (d) Collection distance on microcapsule dimensions.

**Figure S3.** Representative compressive stress-strain curves of ALG hydrogels at different concentrations.

**Figure S4.** Swelling and degradation behaviors of the core–shell hydrogel microcapsules in PBS over 15 days. (a) Swelling ratio of the microcapsules as a function of incubation time. (b) Mass remaining during the degradation process. Data are presented as mean ± SD.

**Figure S5.** (a) Gross view of ovarian cancer specimen. (b) Solid view of ovarian cancer tissue. (c) Optical microscope image of primary ovarian cancer cells. (d) Optical microscope image of primary ovarian cancer cells encapsulated in a hydrogel microcapsule on day D0. (e-h) 3D fluorescence characterization map of tumor spheroids confocal laser imaging encapsulated in hydrogel microcapsules. Scale bars are 200 µm in (c) and 150 µm in (d).


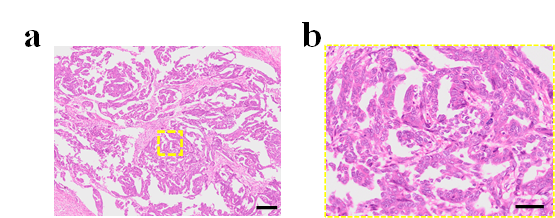


**Figure S6.** H&E staining of patient-derived OC tissue. (a) Low-magnification view; (b) High-magnification view. Scales bars are 200 µm in (a) and 50 µm in (b).


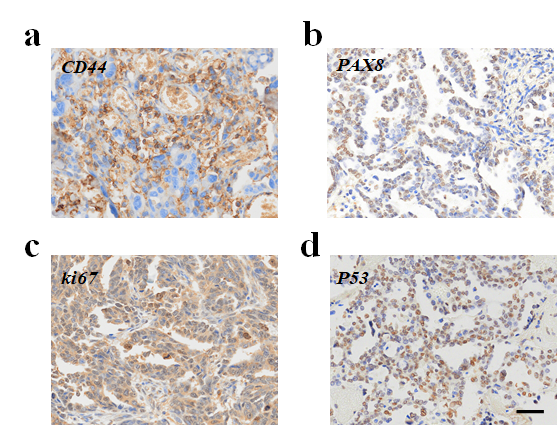


**Figure S7.** Immunohistochemical images of CD44, PAX-8, Ki67 and P53 in ovarian tumor tissues. Scale bar is 50 µm in (d).

**Figure S8.** Optical microscopy images showing the distribution of rhodamine B within the microchannels of the microfluidic concentration gradient generator at different flow rates. In all cases, 100 μM rhodamine B solution was introduced from the left inlet, while blue ink was infused from the right inlet. The flow rates were set to (a) 1.0 μL/min, (b) 2.5 μL/min, and (c) 5.0 μL/min, respectively.

**Figure S9.** (a) Simulation of the simulation process of the concentration gradient formed in the microfluidic drug screening chip. The initial drug concentrations of the left and right inflow solutions were set to 1.0 and 0 mol/m^3^. (b) Simulated process of drug penetration into the tumor-carrying spheroid microcapsules of C10 channel (x-axis is the direction of fluid flow). (c) Simulation process of drug permeation into tumor-carrying spheroid microcapsules with dissected cross section.

**Figure S10.** Levels of apoptosis in three patient-derived tumor spheroids were assessed by fluorescence-activated flow cytometry prior to drug administration.

**Figure S11.** Schematic of the chemotherapy drugs delivery system. Different treatment regimens were applied, including intermittent and continuous drug flows that simulate clinical drug treatment. CBP in blue, PTX was indicated in purple, DTX in green, PLD in brown, and culture medium in light pink. The proportion of the colors in each row represents the reatment time of each drug.

**Figure S12.** Viability of tumor spheroids from three patient-derived groups after 72 h of continuous exposure to single-agent chemotherapy: (a) CBP, (b) PTX, (c) DTX, and (d) PLD.
