# Supplementary figures and images for: Encapsulated Primary Human Ovarian Cancer Cells on Chips for Chemotherapy Drug Evaluation
Source: Research (Wash D C). 2026 Jul 9;9:1313. doi: 10.34133/research.1313 (PMC13346662; doi:10.34133/research.1313)

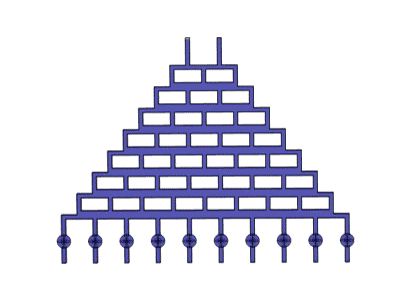

Supplement: Supplementary 1 — Figs. S1 to S12 Movies S1 to S3 [file research.1313.f1.zip › Movie1.gif]

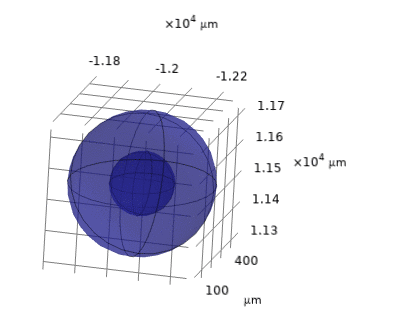

Supplement: Supplementary 1 — Figs. S1 to S12 Movies S1 to S3 [file research.1313.f1.zip › Movie2.gif]

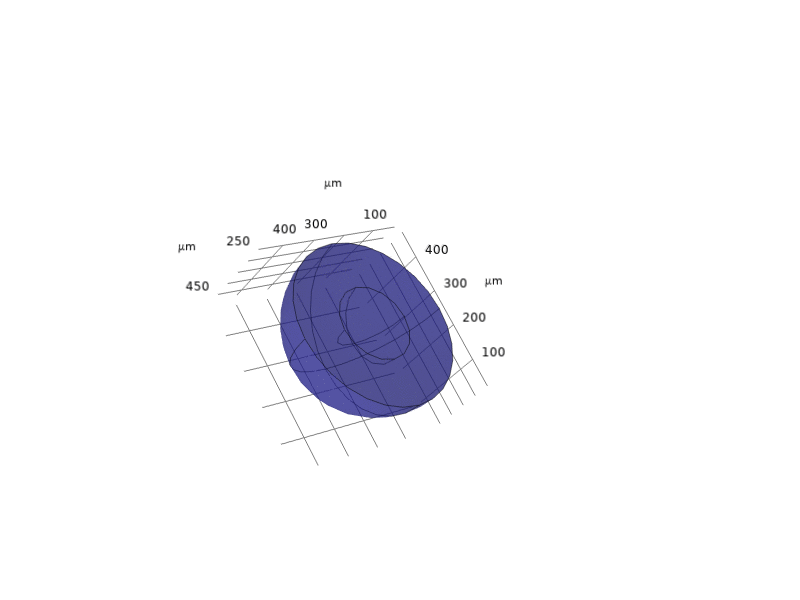

Supplement: Supplementary 1 — Figs. S1 to S12 Movies S1 to S3 [file research.1313.f1.zip › Movie3.gif]
